# Reactive Nanoparticle Composed Bilayers: An Alternate Route Toward the Production of Pt/Al Nanofoils

**DOI:** 10.1002/smll.202501263

**Published:** 2025-07-31

**Authors:** Nishchay A. Isaac, Soumya Biswas, Alper K. Soydan, Arka Mukherjee, Jawahar Rangaraj, Marcel Bohnert, Leslie Schlag, Bardia Aliabadian, Mohammad S.B. Arif, Ji‐Sub Kim, Pedro H.O. Moreira, Juan J. Jiménez, Francisco M. Morales, Peter Schaaf, Andreas Bund, Jörg Pezoldt, Heiko O. Jacobs

**Affiliations:** ^1^ Fachgebiet Nanotechnologie Institut für Mikro‐ und Nanoelektronik Technische Universität Ilmenau Gustav‐Kirchhoff‐Str. 1 98693 Ilmenau Germany; ^2^ Zentrum für Mikro‐ und Nanotechnologien MacroNano Technische Universität Ilmenau Gustav‐Kirchhoff‐Str. 7 98693 Ilmenau Germany; ^3^ IMEYMAT: Institute of Research on Electron Microscopy and Materials University of Cádiz Puerto Real Cádiz 11510 Spain; ^4^ Department of Materials Science and Metallurgical Engineering and Inorganic Chemistry Faculty of Sciences University of Cádiz Puerto Real Cádiz 11510 Spain; ^5^ Fachgebiet für Werkstoffe der Elektrotechnik Technische Universität Ilmenau Gustav‐Kirchhoff‐Str. 5 98693 Ilmenau Germany; ^6^ Fachgebiet Elektrochemie und Galvanotechnik Technische Universität Ilmenau Gustav‐Kirchhoff‐Str. 6 98693 Ilmenau Germany

**Keywords:** energetic material, energy conversion, energy storage, nanofoils, nanomaterials, nanopatterning, spark discharge

## Abstract

Gas‐phase electrodeposition is presented as a nanoparticle‐based route toward the fabrication of Pt/Al bimetallic stacks (self‐propagating reactive system). This approach enables localized self‐assembly of spark discharge‐synthesized sub‐10‐nm Pt and Al nanoparticles on patterned substrates. Precise control over Pt film morphology (porosity) through modulation of spark power and carrier gas flow rate is demonstrated. Porous Pt layers lead to diffused Pt/Al interfaces, which become sharper for densely packed Pt layers. On ignition, the self‐sustained high‐temperature alloy formation reaction wavefronts are recorded. The bimetallic interface strongly influences the Pt/Al reaction kinetics, with three orders of magnitude faster reaction speeds for sharper interfaces. Porous morphologies and hence diffused interfaces are hindered by excessive air gaps and premixed regions, intermediate porosities achieved speeds of 0.012 m s^−1^, and dense morphologies have sharp interfaces with minimal air pockets reaching speeds up to 6 m s^−1^. Selected area (electron) diffraction (SAED) and X‐Ray diffraction (XRD) studies reveal Al_2_Pt and Al_3_Pt_2_ as the dominant alloy phases amongst other intermediate PtAl phases. Furthermore, XRD demonstrates temperature‐dependent facet growth of Pt‐Al alloys. These results prove the critical influence of film morphology on reaction kinetics and emphasize the potential of tuneable Pt/Al bimetallic systems for future energy‐related applications.

## Graphical abstract

Spark ablation‐assisted gas phase electrodeposition is an alternate route toward Pt/Al self‐propagation reactive system synthesis. Pt film morphology‐dependent reaction kinetics is a significant advantage. Low spark power and gas flow rate produce porous films and slow speeds, while high power and flow rate give three orders of magnitude faster reaction speeds up to 6m s^−1^.

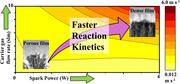

## 1 Introduction

Exothermic reactions in aluminides in the form of nanocomposite materials and multilayer foils are a well‐established process.^[^ ^1^ ^]^ In this phenomenon, the stored internal energy of the components can be released on external stimuli (e.g., ignition) to undergo self‐sustained reactions due to alloy formations of the composed bimetallic stack. The reaction wavefront propagates with velocities up to 90 m s^−1^ as reported in the literature.^[^ ^2^ ^]^ The temperature of the wavefront can reach 1200 K and above for a few microseconds.^[^ ^3^ ^]^ These properties of aluminides make them good candidates for a variety of applications in micro‐joining,^[^ ^4^ ^]^ starting secondary reactions,^[^ ^5^ ^]^ brazing,^[^ ^6^ ^]^ air bags,^[^ ^7^ ^]^ sealing,^[^ ^8^ ^]^ joining refractory materials together^[^ ^9^ ^]^ to name a few examples. The most used aluminide system has been by far the Ni/Al system which first introduced the term *Nanofoils* through patents^[^ ^10^, ^11^ ^]^ from Weihs and Barbee Jr. and now has reached commercial production. However, another system that has seen some growing interest is the Pt/Al system due to its high adiabatic flame temperature^[^ ^12^ ^]^ which makes it a good candidate for low‐thickness heat source applications. Moreover, rapid heat treatment of Pt/Al systems can form a(amorphous)‐Pt_2_Al and Al_5_Pt metastable phases^[^ ^13^, ^14^, ^15^, ^16^ ^]^ which are relatively new, have unique properties not found in conventional Pt/Al alloys, and hence, a primary reason for interest in these systems. Physical vapor deposition (PVD) techniques such as sputtering^[^ ^17^ ^]^ are widely employed for the fabrication of Pt/Al self‐propagating multilayers. It enables precise layering of Pt and Al at nanoscale thicknesses, facilitating controlled diffusion interfaces which are critical for self‐propagating reactions. Other approaches include atomic layer deposition, which uses chemical precursors to deposit layers through successive, self‐limiting gas‐solid reactions.^[^ ^18^ ^]^ Sol‐gel processing provides the synthesis of nanoscale energetic materials through solution‐based reactions that yield uniform precursor dispersions.^[^ ^19^ ^]^ Electrodeposition, involving the reduction of Pt and Al ions onto substrates under controlled conditions, is also being employed for its simplicity and cost‐effectiveness.^[^ ^20^ ^]^

Various parameters on the micro/nanoscopic scale affect the thermodynamics and kinetics of the self‐propagation reaction: (i) bilayer structure evolution^[^ ^21^ ^]^ (ii) reaction mechanism^[^ ^22^ ^]^ (iii) bilayer architecture^[^ ^23^ ^]^ (iv) stoichiometry of the multilayer stack^[^ ^24^ ^]^ (v) heat of reaction^[^ ^25^ ^]^ (vi) bilayer spacing and dimension^[^ ^26^ ^]^ and (vii) nanoparticle sizes.^[^ ^27^ ^]^ The combined effect of all these factors is manifested macroscopically in the wavefront propagation speeds and reaction temperature of these reactive materials. By tuning these stack parameters, one can tune the wavefront properties depending on the application. For instance, by adjusting the component molar ratio (e.g., Ni/Al, Pt/Al), the front velocity can be changed.^[^ ^28^ ^]^ Zhu et al. have demonstrated this for the Ni/Al system:^[^ ^29^ ^]^ a 1:1 molar ratio gives higher reaction speeds and combustion temperatures as compared to the 1:5 Al/Ni and 3:1 Al/Ni systems. Smaller molar ratios minimize heat loss and phase transition, leading to faster reaction kinetics and increased flame temperature. Wavefront velocities are also a function of microstructure.^[^ ^30^ ^]^ In powder‐based systems, reactivity is enhanced through the bimetallic concentrations. As demonstrated by Chen et al.^[^ ^31^ ^]^ on increasing the Ni content in the Al matrix in a cold spray process, the continuous phase of Al is partially replaced by the Ni particles intermediate phase where the bimetallic interfaces grow. Increased contact points between Ni‐Al are promoted by smaller particle sizes. As a result, the ignition threshold and reaction efficiency can be tuned. Bilayer thickness and design are other important factors for reaction kinetics. With increasing bilayer stack thickness, more energy is required for ignition. The amount of pre‐mixing increases with decreased bilayer thickness. Pt/Al systems with individual bilayers below 20 nm in thickness show no self‐propagation reaction owing to pre‐ignited alloy formation.^[^ ^30^ ^]^ An external route to affect the energetic system chemistry is substrate design for multilayer deposition.^[^ ^32^ ^]^ Deposition of such energetic systems on Si needle‐shaped substrates as compared to flat substrates, promotes the formation of multiple stable phases as compared to a single phase.^[^ ^33^ ^]^ This change in alloying reactions is a result of additional atomic diffusion promoted by the needle‐like structures. As a result, the reaction kinetics are affected. Moreover, substrates act as heat sinks which provide a hindrance to wavefront propagation.^[^ ^34^ ^]^ While self‐propagation reactions are quenched on substrates with high thermal conductivity, reactions can occur as the heat loss is controlled on low‐thermal‐conductivity substrates.^[^ ^35^ ^]^

In this work, different from the current state‐of‐the‐art approaches for the fabrication of multilayer bimetallic stacks, a gas phase method is showcased. Spark discharge is a well‐established process used for nanoparticle synthesis and has demonstrated success in applications of gas sensors,^[^ ^36^ ^]^ smart textiles,^[^ ^37^ ^]^ vias,^[^ ^38^ ^]^ high‐entropy alloys,^[^ ^39^ ^]^ interconnects^[^ ^40^ ^]^ to name a few. Spark discharge‐assisted nanoparticle synthesis is used in this work, to produce positively charged nanoparticles below 10 nm in size. The synthesized nanoparticles (i.e., Pt and Al) are transported toward the substrate by a carrier gas. The positively charged nanoparticles are self‐assembled into linear openings on a negatively biased substrate. By sequentially alternating the source of nanoparticles, Pt/Al bilayer stacks are fabricated. This approach is unique and has not been demonstrated before to form multilayer stacks. Pt/Al multilayer stack lines can be ignited to give a self‐sustaining reaction wavefront which is characterized in this article. It is demonstrated that the wavefront velocity of the synthesized stacks can be tuned over three orders of magnitude by efficiently controlling deposited layers morphology and consequently the Pt/Al bimetallic interface. By using different values of spark power and carrier gas flow rate, porous or dense morphologies of deposited Pt layers were obtained while keeping the Al morphology constant. Porous films give a diffused bimetallic interface which becomes sharper with denser morphologies. All the samples have been subjected to the same ignition conditions, and high‐speed videography has been recorded simultaneously. The tunability of the wavefront propagation velocity is discussed in detail and supported by systematic investigations with transmission electron microscopy (TEM) experiments, X‐ray diffraction (XRD) studies, and high‐speed videography.

## 2 Results And Discussions

**Figure 1:**
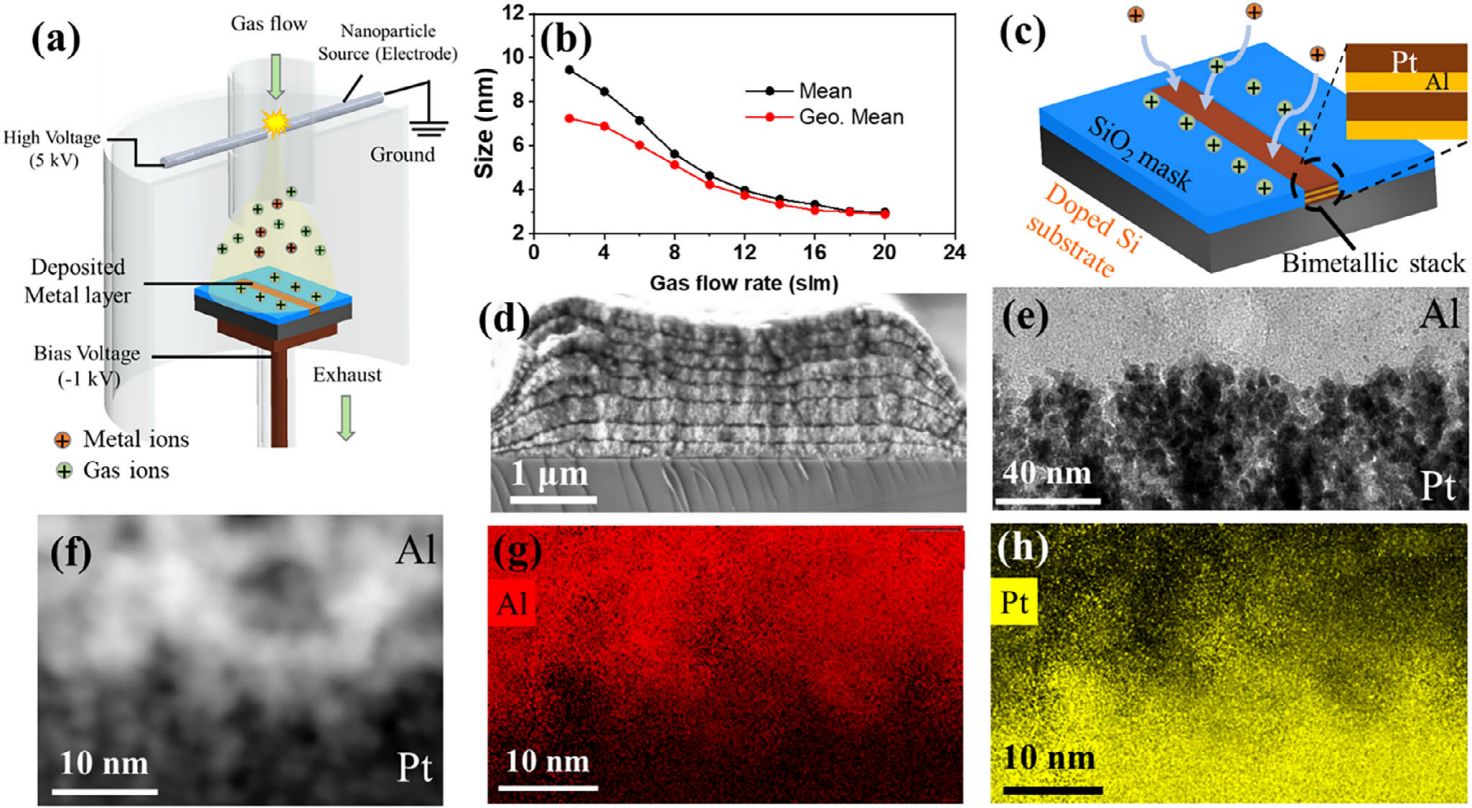
Pt/Al multilayer stack fabrication results. In a) schematic diagram of the spark discharge acrylic reactor is presented with N_2_ gas flow and exhaust. Positively charged nanoparticles are carried toward the biased substrate with N_2_ gas flow. b) The Size dependency of generated nanoparticles with the applied N_2_ flow rate, higher gas flow leads to smaller particles. c) Schematic diagram showing how the generated nanoparticles are deposited into linear openings on a dielectric (SiO_2_) patterned and electrically biased Si substrate. Arrows indicate the fringing fields, which arise from the positive charges decorated on the dielectric, increasing the electrical potential of the dielectric during the experiment. The field directs the positive nanoparticles into the linear openings. d) 8‐Bilayer stack cross‐section SEM image to observe the film morphology. e) The cross‐section observed in a STEM micrograph highlighting the diffused Pt/Al interface. The interface is further zoomed in to a scale of 10 nm f). EDX mapping was performed on an area at the diffused interface as shown here. The corresponding element net counts map was obtained, showing the presence of the mixture of both g) Al and h) Pt.

The synthesis of nanoparticles is carried out at room temperature and 1 bar pressure inside a cylindrical acrylic reactor as shown in **Figure** **1**a. An inlet houses two metal electrodes (interelectrode gap of 1 mm), one placed at a high voltage of 5 kV (anode) and the other one grounded (cathode). A flow of nitrogen gas (N_2_) is maintained through the gap. The strong electric field in the gap ionizes the gas, initiating the plasma, and metal vapor is thus produced through cathode erosion.^[^ ^41^, ^42^, ^43^ ^]^ Subsequently, the metal vapor quenches in the flow of carrier gas and forms nanoparticles which are positively charged. The residence time of the metal atoms in hot regions of the plasma and the quenching effect of the carrier gas flow decide the primary particle size and the degree of agglomeration‐aggregation of synthesized nanoparticles.^[^ ^44^ ^]^ As formulated by Lehtinen et. al.^[^ ^45^ ^]^ heat is generated due to particle collisions in regions close to plasma, which leads to coalescence and hence particle size growth until the system is quenched. On increased quenching, the cooling time is faster than the collision time amongst atoms/particles and this defines the primary particle size. Therefore, it is observed that at high gas flow rates, smaller primary particle sizes are observed.^[^ ^38^ ^]^

In our setup, by adjusting the gas flow rate through the spark between 2 standard liters per minute (slm) and 20 standard liters per minute (slm), the mean size of produced nanoparticles can be tuned (Figure 1b). To determine the average size of particles, they are collected on a Cu quantifoil and observed under a Gemini 500 Zeiss scanning electron microscope (SEM) in transmission mode. The size distributions are determined from 75 randomly selected particles on the grid, and a log‐normal distribution is observed. From this distribution, the average particle sizes are calculated. Detailed calculations for Pt nanoparticles are provided in Figure S1 (Supporting Information). The ionized gas atoms outnumber the nanoparticles produced by an order of magnitude as determined experimentally.^[^ ^46^ ^]^ Since the gas ions are positively charged, this charge is imparted to most of the nanoparticles produced.^[^ ^47^ ^]^ The particles are incident on a doped Si‐chip, which is coated with thermally grown SiO_2_.

The charged particles are locally self‐assembled on pre‐determined locations on the substrate through the funnel effect of gas phase electrodeposition.^[^ ^48^ ^]^ In short, gas ions with mobility higher than that of the metal nanoparticles decorate themselves on the dielectric and create fringing fields when the substrate is negatively biased. These fields direct the slow‐moving nanoparticles into the openings (check Figure 1c). In these trenches, charged species dissipate their charges and form layered depositions. By periodically changing the source of nanoparticles between Pt and Al, a bimetallic multilayer stack (Figure 1d) can be achieved. To study the bimetallic interaction between Pt and Al at multiple interfaces across the stack, a focused ion beam (FIB) system is used to cut and thin lamellae that could be investigated by TEM. Contrary to the usual sharp interfaces observed in vapor‐deposited films, nanoparticle‐based films show a relatively diffused interface between Pt and Al (Figure 1e). The extent of diffusion between the layers is a function of particle size, gas flow rate, substrate bias, and spark power. By designing the deposition experiment with each of these factors, the bi‐metallic interface can be tuned, and the intermetallic diffusivity can be controlled. In the as‐deposited films, elemental mapping experiments, which are obtained by energy‐dispersive X‐ray spectroscopy (EDX), are done on a scale of 10 nm to determine the degree of intermixing between the two components. As shown in Figure 1f, at 4 W spark power, 4 slm N_2_ gas flow rate, and ‐1 kV substrate bias, a relatively diffused Pt/Al interface is observed under scanning‐transmission electron microscopy imaging conditions (STEM) prior to the aforementioned EDX experiments. Element mapping in Figure 1g,h at one of the interfaces shows the distribution of Pt (yellow) and Al (red) nanoparticles. The comparison of both maps allows to roughly estimate a diffusion length of ≈25 nm, since the signals of both elements can be found at either side of the Al/Pt interface. In the inset images of Figure 1g,h, the selected area electron diffraction (SAED) patterns, which plot the behaviour in the reciprocal space of Al‐rich and Pt‐rich regions comparable to the ones shown in these images, are shown. The *d*‐spacing calculations for the diffraction rings found at the Pt layer are provided in Figure S2 (Supporting Information), whereas explanations on how crystallographic calculations were carried out for this article is compiled in the experimental section and in Figure S3 (Supporting Information).

**Figure 2:**
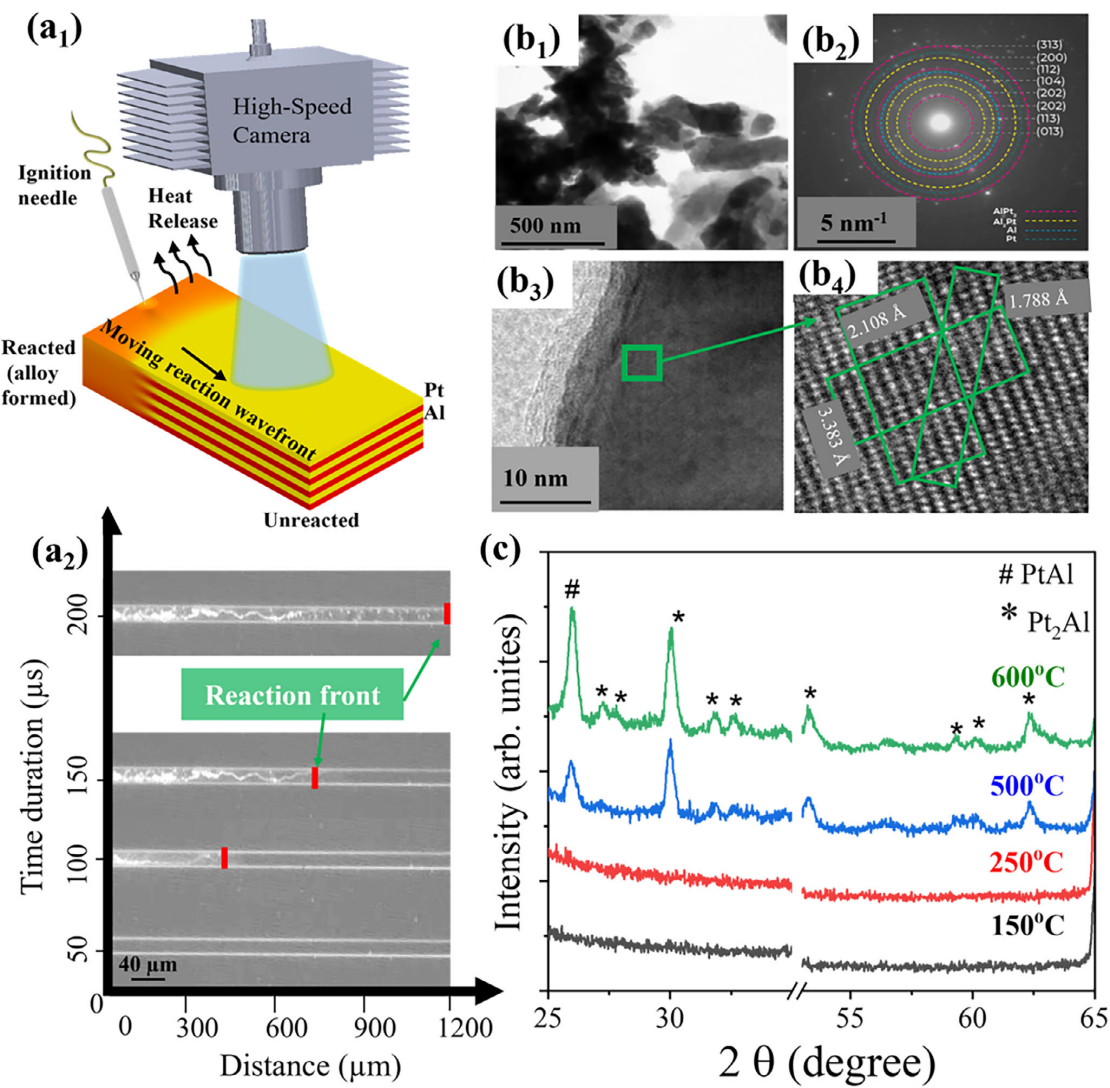
Pt/Al self‐propagation reaction videography and material analysis. (a_1_) schematic diagram of the high‐speed videography setup installed on a stereomicroscope to observe Pt/Al multilayer stack self‐propagation reaction. A tungsten needle at the far end ignites the stack. Unreacted Pt (in yellow) and Al (in red) multilayer stack on alloy formation (in orange) is shown schematically. The reaction wavefront propagation from left to right is schematized by the black arrow. (a_2_) Temporal screenshots of the wavefront propagation post ignition are shown for a 15‐µm wide line pattern with the red line denoting the reaction wavefront used for the reaction speed calculation. (b_1_) is a HAADF image of the formed alloy after the reaction. (b_2_) SAED pattern of the formed alloy confirming the polycrystalline nature by the presence of Pt‐Al alloy planes. (b_3_) Not zoomed HRTEM image of the reacted alloy showing their lattice arrangements. The area of interest in green contains signals magnified in (b_4_), which is another HRTEM image to confirm the presence of multiple planes. The HRTEM image of the marked part confirms the presence of the different planes corresponding to Al_2_Pt. (c) XRD patterns in the 2θ angle range of 25°–34° and 51°–65°, performed on a large area‐deposited Pt/Al multilayer film and annealed at different temperatures.

On ignition, the Pt‐Al multilayer system forms a self‐sustaining reactive system observable under a high‐speed videography setup. The recording setup is shown in **Figure** **2**a_1_ schematically. A high‐speed video camera is installed on a vertical stage of the stereo microscope to focus on the Pt/Al multilayer stack on the substrate. A high‐voltage tungsten needle initiates 60 mW sparks at one end of the deposition. A reaction wavefront can be seen propagating from left to right as indicated by the arrow and can be captured on the camera at 5000 frames per second (https://cloud.tu‐ilmenau.de/s/ENci8FSSdSJpWHE). Temporal screenshots (Figure 2a_2_) are used to compute the reaction velocity. In the given sample, a wavefront velocity of 6 m s^−1^ is observed post‐reaction. Post‐reaction products are analysed by high‐angle annular dark field (HAADF) images as shown in Figure 2b_1_. Moreover, when analysed by SAED, the resulting materials show concentric rings as presented in Figure 2b_2_. The *d*‐spacing calculations (see experimental section) prove the existence of Al_2_Pt (112), (202), (113) and Al_3_Pt_2_ (313), (104), (013) alloy lattice planes, along with signals originating from remnants of unreacted Pt and Al. For further confirmation, a closer look with TEM is made under high‐resolution conditions (HRTEM, Figure 2b_3_), which make lattice arrangements visible. The green square in Figure 2b_3_ is further magnified in a separate HRTEM image (Figure 2b_4_) which shows lattice spacings of ≈2.11, 3.38, and 1.79 Å corresponding to Al_2_Pt alloy formation projected along the [112] zone axis. It is interesting to mention that the morphology of reacted Pt/Al bimetallic stacks is dependent on the ignition temperature ramp rates. For gradual temperature ramps (≈1 °C s−^1^), multilayer stacks tend to react slowly, transforming the multilayer stack into a bimetallic mixture with no considerable reaction wavefront observation. On the other hand, as rapid thermal ignition (100 µs spark ignition event) is carried out, it results in short duration steep temperature ramps (≈5 °C µs^−1^). Layers react aggressively, making them extremely porous. FIB cuts for such films show spherical‐featured morphology of the Pt/Al reacted stack (Figure S4, Supporting Information) cross section observed by SEM. This could be due to surface tension.^[^ ^49^ ^]^ The Pt/Al stack likely enters a liquid phase during the reaction; however, this phase is expected to last only for a moment due to the rapid nature of self‐propagation. It is well‐recognized that surface tension drives small amounts of liquid to form rounded shapes.^[^ ^50^ ^]^ Following this short‐lived liquid state, the reacted Pt‐Al quickly solidifies. In our previous work,^[^ ^51^ ^]^ differential scanning calorimetry (DSC) studies have shown heat evolution of 150 mW g^−1^ ≈135 °C and 12 mW g^−1^ ≈400 °C. To investigate alloy formation during multiple heat evolution events, temperature‐dependent X‐ray diffraction (XRD) is conducted at 150, 250, 500, and 600 °C. Figure 2c shows the results of these measurements. Consistent with previous findings, the presence of cubic Al_2_Pt alloy (65‐2983 PDF), low‐intense orthorhombic AlPt_2_ (29‐0069 PDF), and orthorhombic Al_6_Pt (47‐0981 PDF) along with minor Pt peaks in the grazing angle (2θ) range of 32°–51° indicate the formation of the Pt/Al alloy due to the exothermic reaction ≈135 °C. Furthermore, the XRD measurements executed at 500 and 600 °C reveal the evolution of multiple new peaks corresponding to PtAl and AlPt_2_ alloys formation within the grazing angle range of 20°–32° and 51°–75° as depicted in Figure 2c. These observations confirm the influence of the exothermic DSC peaks that are observed ≈400^ °^C. Additionally, XRD measurement reveals that, above 500 °C, the sample undergoes a disproportion reaction. This is evidenced by an enhancement in the relative intensity of the AlPt_2_ peak (≈30°) compared to the PtAl peak (≈26°). Consequently, during ignition, the sudden elevated temperatures facilitate disproportionation reactions. This leads to the conversion of PtAl into either Al_3_Pt_2_ or Al_2_Pt, resulting in the absence of PtAl planes in TEM analyses.

**Figure 3:**
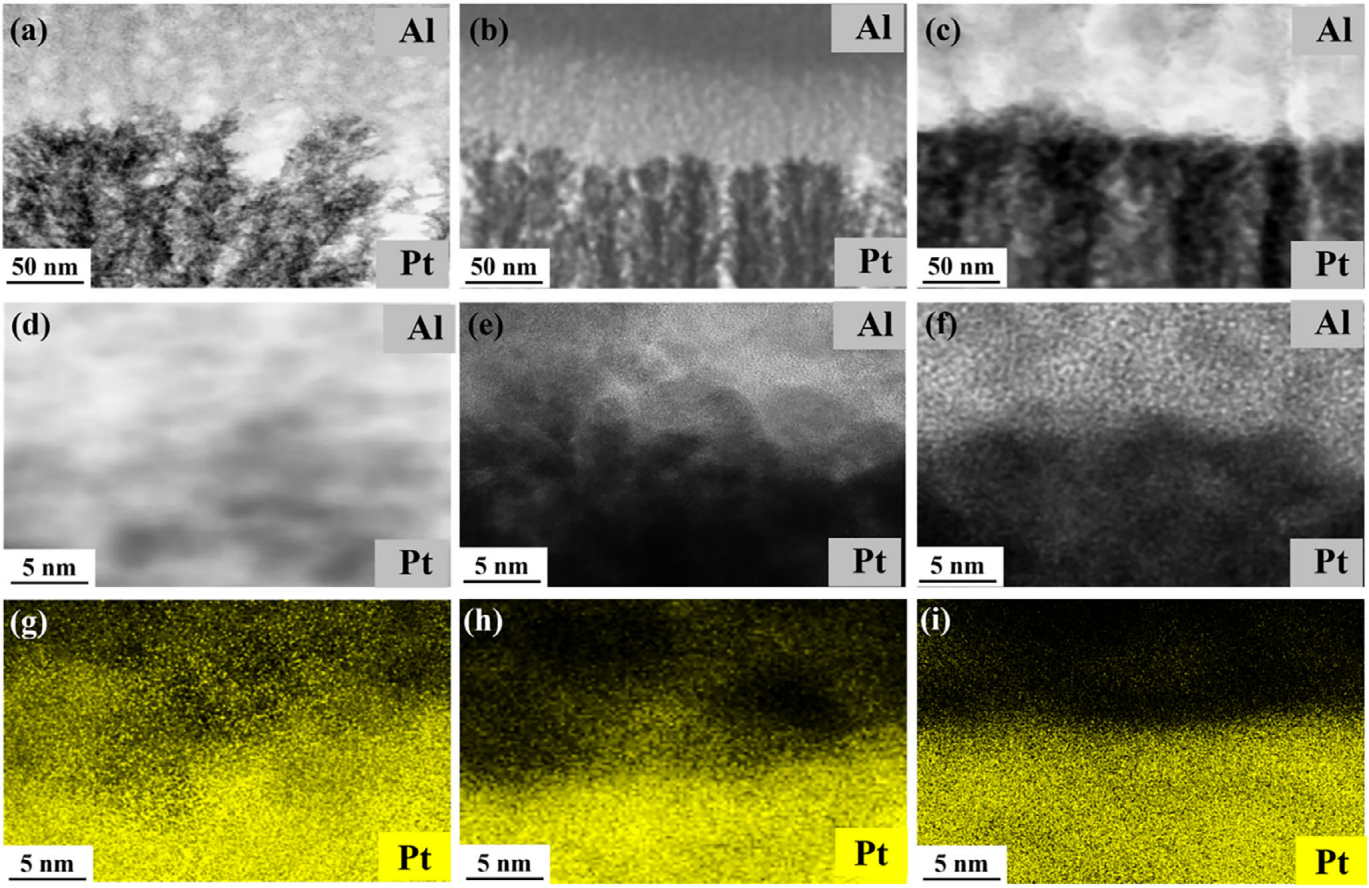
(S)TEM images from spark parameter‐dependent morphology of Pt layers. In a) 2 W spark power with 4 slm carrier gas flow rate was used, b) 4 W with 6 slm, and c) 4 W with 10 slm. (d–f) show the corresponding Pt‐Al interfacial morphology on a scale of 5 nm. As the Pt layer becomes denser, the interfacial spread decreases as shown clearly by EDX mapping. In g) with a porous morphology, the Pt (yellow) nanoparticles spread is above ≈25 nm. In h) with intermediate morphologies obtained at higher spark power and high gas flow rate, the spread decreases to ≈15 nm, and in i) with high spark power and gas flow rate of 10 slm, the spread even further decreases to below 5 nm, giving a sharp Pt‐Al interface.

The self‐sustaining reaction kinetics for stacks fabricated by gas phase electrodeposition depends strongly on the morphology.^[^ ^52^, ^53^ ^]^ Hence, an efficient control of the structure of the Pt/Al bimetallic interface forms one of the notable advantages of using this method for such self‐sustaining reactive systems. The layer porosity can be controlled by varying the spark power and carrier gas flow rate during synthesis.^[^ ^46^ ^]^ At low gas flow rate of 4 slm and low power of 2 W, porous morphologies are obtained (**Figure** **3**a) and with increasing the power to 4 W and flow rate to 6 slm, the morphology becomes moderately dense (Figure 3b). On further increasing the given parameters (i.e., 4 W and 10 slm N_2_), high‐density films can be achieved (Figure 3c). There are various factors responsible for this parameter‐dependent film morphology. The produced nanoparticle trajectory inside the reactor from the synthesis zone to the deposition zone is initially governed by the carrier gas flow dynamics, and close to the substrate it is governed by the substrate bias.^[^ ^54^ ^]^ As the flow rate increases, the fluid dynamics dominated zone increases, leading to a diminished effect of stochastic processes (e.g., diffusion) which cause dendritic growth. Hence, dense layer growth is observed. Moreover, an increased flow rate decreases nanoparticle size, leading to more compact films. In our experiments, the particle sizes at 2 slm have been observed to be ≈9.5 nm, which has decreased to 3 nm at flow rates of 20 slm. Additionally, as the flow rate is increased the dilution levels of produced nanoparticles in carrier gas molecules increases. Consequently, the aggregation/agglomeration of the produced nanoparticles is decreased, leading to film growth in a relatively more dense fashion. Kinetic energy of the particles also plays a major role. It was highlighted by Schlag et al.^[^ ^46^ ^]^ that, as the spark power is increased, larger particles impart a higher kinetic energy to the substrate on deposition. This increases the substrate temperature, which has been measured for the given experiments.^[^ ^46^ ^]^ This increased substrate temperature leads to more order and increases the density of the deposited films. The tuneable morphology of Pt layers from porous to dense layers is observed on a fine scale of 5 nm. As shown in Figure 3d–f, as we move from left to right, both the power and gas flowrate increase. Pt films became more compact, which has a direct effect on the intermetallic interface. This is better observed by resorting to EDX element mapping again. As shown for Pt (yellow) in Figure 3g, the interface is diffused with a Pt spread of ≈25 nm if the scale is used as a guide. For intermediate packing densities of Pt films, the spread is lowered to ≈15 nm (Figure 3h). At higher flow rate and power (Figure 3i), the Pt‐Al interface becomes sharp with a minimal Pt interfacial spread below ≈5 nm.

**Figure 4:**
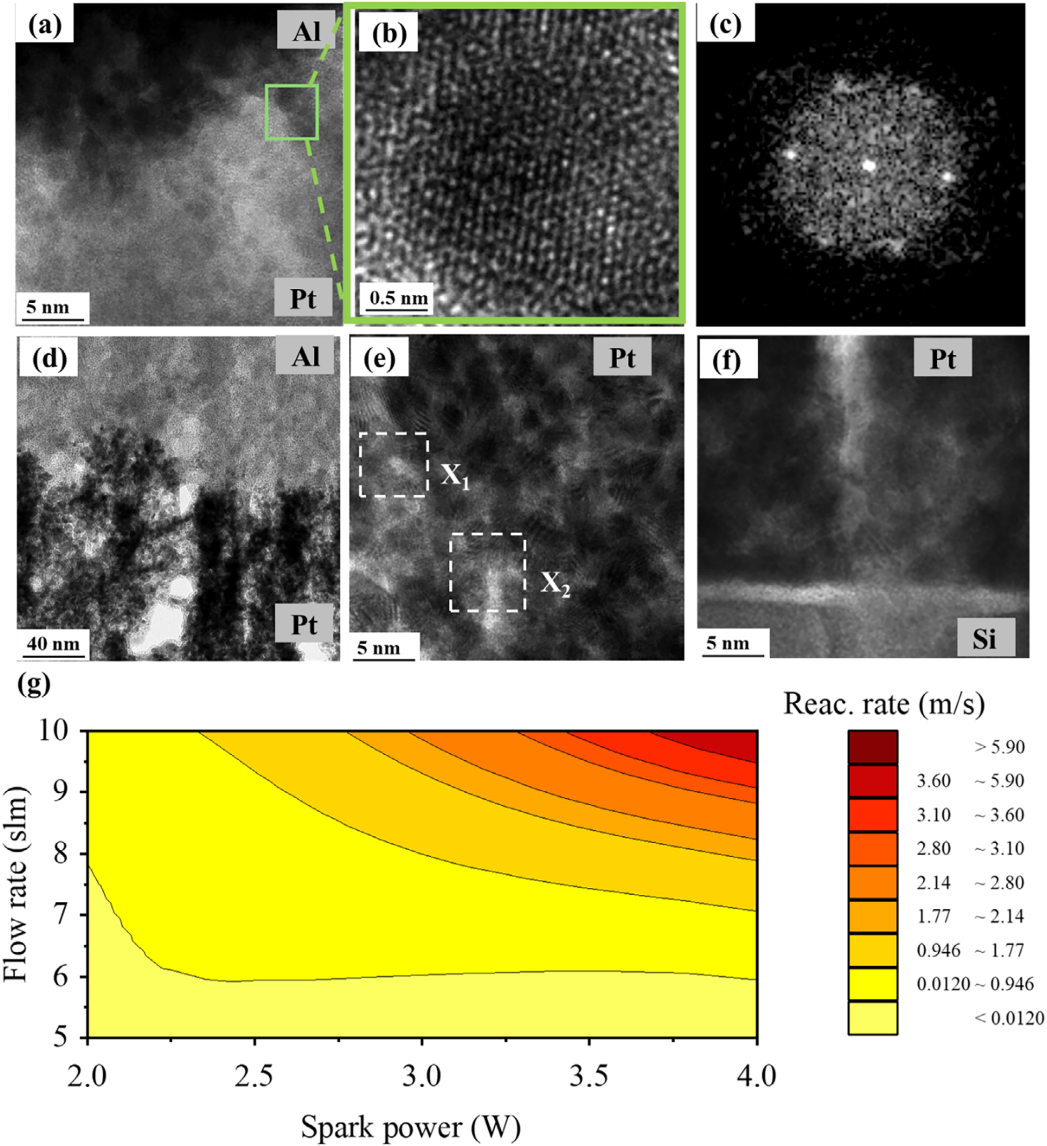
Morphology‐dependent reaction wavefront speeds results. a) HRTEM image showing the area of interest (green square) to check the possibility of pre‐mixed PtAl intermetallic compounds. b) Additional HRTEM micrograph at higher magnification to analyse this interfacial area. c) FFT retrieved from the previous HRTEM image showing a measurable hexagonal arrangement of bright spots. d) TEM image with incorporated air gaps above 100 nm in thickness in Pt layers. e) Pt layers observed by HRTEM on a scale of 5 nm, showing two regions (X_1_ and X_2_) with air gaps above 5 nm in size. f) HRTEM micrograph depicting several airgaps extended across the height of the Pt layer and appearing as pinholes. g) Wavefront velocity results of 30 different experiments presented as a trend map.

Bilayer systems often form premixed layers in their as‐deposited phase.^[^ ^52^ ^]^ In the given system, as the Pt/Al contact points increase owing to porosity, the premixed layers become prominent. This feature is studied again by HRTEM. The small regions along the Pt‐Al interfaces were inspected by retrieving fast Fourier transforms (FFTs) from the corresponding HRTEM images. Starting at a region like the one shown in **Figure** **4**a, HRTEM images of local features are eventually obtained (Figure 4b). The FFT is thus retrieved from the images of these local areas, which sometimes led to hexagonal arrangements of reflections (Figure 4c). By measuring the angles of the bright spots and the spacing between them, the *d*‐spacing values can be computed. By using the tools cited in the experimental section, diffraction patterns were calculated and could be indexed to AlPt intermetallics, primarily Al_3_Pt_2_ and Al_2_Pt as the most likely candidates.

Another salient feature of porous Pt films is the trapping of air gaps in between the nanoparticles. Since the self‐sustaining reaction propagates due to the thermal conduction in the deposited layer, the incorporation of air gaps, which act as heat sinks, can thus be used to slow down or stop the reaction. Air gaps can be large, spanning sizes above 100 nm as shown in Figure 4d. However, for denser morphologies, it can be very small in size, such as below 5 nm as shown in X_1_ and X_2_ regions marked in Figure 4e. Several air gaps can also extend as pinholes in the complete Pt layer till the Si growth substrate (Figure 4f).

The reaction wavefront velocities have been calculated for 30 different experiments. The total thickness of the bimetallic stack was kept fixed at 2.5 µm, and with the length of 1000 µm. Each stack consists of 4 bilayers. Pt film morphologies have been tuned by varying the spark power and N_2_ gas flow rate, keeping the deposition parameters fixed for Al layers. The details of the deposition parameters are tabulated in **Table** **1**.

**Table 1:** The deposition parameters for different categories of bilayer stacks:

Sample categories	Deposition Flow Rate for Pt [slm]	Deposition Spark Power for Pt [W]	Deposition Flow Rate for Al [slm]	Deposition Spark Power for Al[W]
Porous	4	2	10	4
Moderately dense	6	4		
Dense	10	4		

Based on the experiments, a trend can be seen by plotting it as a contour colour map in Figure 4g. The detailed experimental plot is shown in Figure S5 (Supporting Information). For low spark power and gas flow rates, wave front velocities are slow and can be tuned up to three orders of magnitude at high spark power and carrier gas flow rates. Porous Pt film morphologies give dendritic films which incorporate > 100 nm air gaps and pinholes, which hinder the propagation of the reaction wavefront as they are heat sinks. Moreover, increased contact points between Pt‐Al cause premixed regions. As the spark power and gas flow rate are increased, the Pt films tend to become densely packed (i.e., air gaps below 5 nm in size) and Pt/Al interfaces become sharper with a low percentage of premixed regions. The wavefront velocity therefore, speeds up by almost 3 orders of magnitude up to 6.1 m s^−1^. Hence, by controlling the morphology of the bilayers, the wavefront velocities can be tuned depending on the energy applications where these stacks are to be used.

## 3 Conclusion

This study facilitates the localized self‐assembly of sub‐10 nm synthesized nanoparticles of Pt and Al, enabling material‐efficient growth of nanoparticle‐based metallic layers. The Pt/Al multilayers exhibit distinct morphologies and a tuneable intermetallic interface ranging from diffused to sharp, signifying programmable transitions between the two metals. Pt/Al bimetallic stacks on spark ignition demonstrate a rapid, self‐sustained high‐temperature alloy formation reaction system. Using high‐speed videography, the reaction dynamics of deposited multilayer stacks are analysed. Porous Pt film morphologies showcase dendritic films and extensive Pt‐Al premixed layers, which act as a hindrance to self‐propagation reaction wavefronts. At moderate Pt film porosities, reaction speeds average 0.012 m s^−1^, while dense morphologies with minimal air pockets, a sharp Pt‐Al interface, and negligible premixed areas exhibit 3 orders of magnitude faster speeds, up to 6 m s^−1^.

Post‐reaction analysis via XRD identified Al_2_Pt and Al_3_Pt_2_ as the primary alloy phases. DSC studies from prior work^[^ ^55^ ^]^ have revealed two exothermic peaks during alloy formation, occurring at 135 and 400 °C, while XRD at different temperatures (100 °C–600 °C) correlate the exothermic peaks to the presence of multiple intermediate alloy phases (e.g., PtAl). Cross‐sectional FIB imaging of the reacted films reveals a surface tension‐controlled morphology, indicative of an intermediate liquid phase during alloy formation.

The observed morphology‐dependent reaction kinetics of the Pt/Al system serve as a preliminary display of the film architecture‐alloy formation correlation. A comprehensive investigation is nevertheless required to establish detailed relationships between nanoparticle size, multilayer stack dimensions, and ignition temperatures, which remains an active area of research. Such advancements could pave the way for the development of Pt‐Al bimetallic layers tailored for future energy applications.

## 4 Experimental Section

### Nanoparticle Generation

Nanoparticles were synthesized in an MBRAUN glovebox with <0.1 ppm O_2_ and <0.1 ppm H_2_O. A spark generator was composed of a cylindrical acrylic reactor having a 6 mm inlet (Outer Diameter) at the top. The inlet was connected to a cylindrical acrylic tube that had two consumable electrodes (≈1 mm apart). One of the electrodes was grounded while the other was set to 5 kV direct current (DC) voltage. The electrodes were made of Pt and Al, purchased from Advent Research Materials‐UK. To induce spark discharges between electrodes, a flow of 10 slm gas was passed through the tube. To collect nanoparticles, a substrate was placed 1.5 cm below the discharge region and set to a DC voltage of −1 kV.

### Substrate Preparation

The 1 µm wet silicon dioxide mask was transferred to the surface of silicon wafers using a maskless aligner, MLA150 from Heidelberg Instruments. Acetone, isopropyl alcohol, and de‐ionized water were used for sequential cleaning of the wafers. Lithography was done with AZ1518 photoresist (MicroChemicals GmbH), and after development, oxide was removed using buffered oxide etch (BOE) 7:1 solution.

### FIB Preparations

ZEISS FIB Nanoanalytik Auriga 60 was used to prepare FIB lamellae. A magnetic platinum‐carbon layer was deposited prior to thinning the lamella with a gallium ion beam. The lamella was placed onto Omniprobe copper grids (Plano GmbH J420). FIB thinning was performed from an initial thickness of ≈900 nm to a final value of ≈100 nm. The initial and final voltages and currents during this process were 30 kV, 600 pA, and 5 kV, 240 Pa, respectively.

### Grid‐Based TEM Sample Preparation and Particle Size Calculation by STEM

Reaction products are dissolved in ethanol and added dropwise onto a TEM grid. The solvent dries up and the material is stuck to the lacey carbon grids, which is analysed afterward using a Thermo Scientific Talos F200X microscope operated at an acceleration voltage of 200 kV. The same microscope was used to study the FIB lamellae. Under scanning‐transmission conditions, high‐angle annular dark‐field (HAADF) images were registered using a camera length of 125 mm. SAED imaging was registered under TEM imaging conditions at variable camera lengths depending on the case (either 840 mm or 1.10 m).

A similar TEM grid was used to collect micrographs by using STEM. The calculation of the mean, geometric mean, and standard deviation of the size of the produced particles from those images are discussed thoroughly in Figure S1 (Supporting Information). The STEM micrographs are shown in Figure S1.1 (Supporting Information).

### Methods for Crystallographic Calculi

Throughout this work, several intermetallic materials are mentioned together with pure Pt. In order to obtain their theoretical lattice spacings and angles, Eje‐Z software^[^ ^56^ ^]^ as been used as a supporting tool. This was also used the same software to generate electron diffraction patterns, which allowed this to make comparisons with this experimental findings in FFTs retrieved from HRTEM images. A book on electron diffraction is also employed as guidance to calculate *d*‐spacings and check some possible arrangements of spots.^[^ ^57^ ^]^ To use Eje‐Z successfully and carry out sensible comparisons with the experimental results, this was needed to gather some key information from several sources depending on the considered materials. In this sense, for Al_2_Pt, the lattice constants come from the following article,^[^ ^58^ ^]^ whereas the atomic coordinates come from the Springer Materials database (entry 1940281).^[^ ^59^ ^]^ For Al_3_Pt_2_, the lattice constants are extracted from another work^[^ ^60^ ^]^ and the atomic coordinates were found in the Materials Project website (entry 10905). Information on pure Pt for its simulation comes from another book.^[^ ^61^ ^]^

## Conflict of Interest

The authors declare no conflict of interest.

## Supporting information



Supporting Information

## Data Availability

The data that support the findings of this study are available from the corresponding author upon reasonable request.
