# Supplementary material for: Reactive Nanoparticle Composed Bilayers: An Alternate Route Toward the Production of Pt/Al Nanofoils
Source: Small. 2025 Jul 31;22(12):e01263. doi: 10.1002/smll.202501263 (PMC12934383; doi:10.1002/smll.202501263)
Supplement: Supplementary file 1 — Supporting Information [file SMLL-22-e01263-s001.docx]

Supporting Information

**Reactive nanoparticle composed bilayers: An alternate route towards the production of Pt/Al Nanofoils**

N.A. Isaac^1,2^ S. Biswas^1,2,^ A.K. Soydan^1,2^, A. Mukherjee^1,2,^ J. Rangaraj^1,2^, M. Bohnert^1,2^, L. Schlag^1,2^, B. Aliabadian^1,2^, M.S.B. Arif^1,2^, J. Kim^1,2^, P.H.O. Moreira^1,2^, J.J. Jimenez^3,4^, F.M. Morales^3,4^, P. Schaaf^2,5^, A. Bund^6^, J. Pezoldt^1,2^, H.O. Jacobs^1,2 *^

**Supporting Information S1**

Particle morphology and size distribution were assessed by collecting deposited particles on a TEM grid and performing Scanning Transmission Electron Microscopy (STEM). Micrographs were acquired, and a representative visible area was selected from each image. Within this area, 75 particles were randomly chosen, and their diameters were determined using ImageJ software, referencing the scale bar embedded in the STEM images for accurate calibration. The compiled particle size data, recorded in an Excel spreadsheet, were used to compute the arithmetic mean, geometric mean (GM), and standard deviation (SD). Subsequently, the measured particle sizes were plotted as a log-normal distribution to independently verify the calculated mean and SD. For clarity, two examples of these calculations are demonstrated for particles generated at gas flows of 4 standard liters per minute (SLM) and 8 standard liters per minute (cf. **Figure S1.1**), with tabulated results (**Table S1**) for all investigated flow rates provided thereafter.


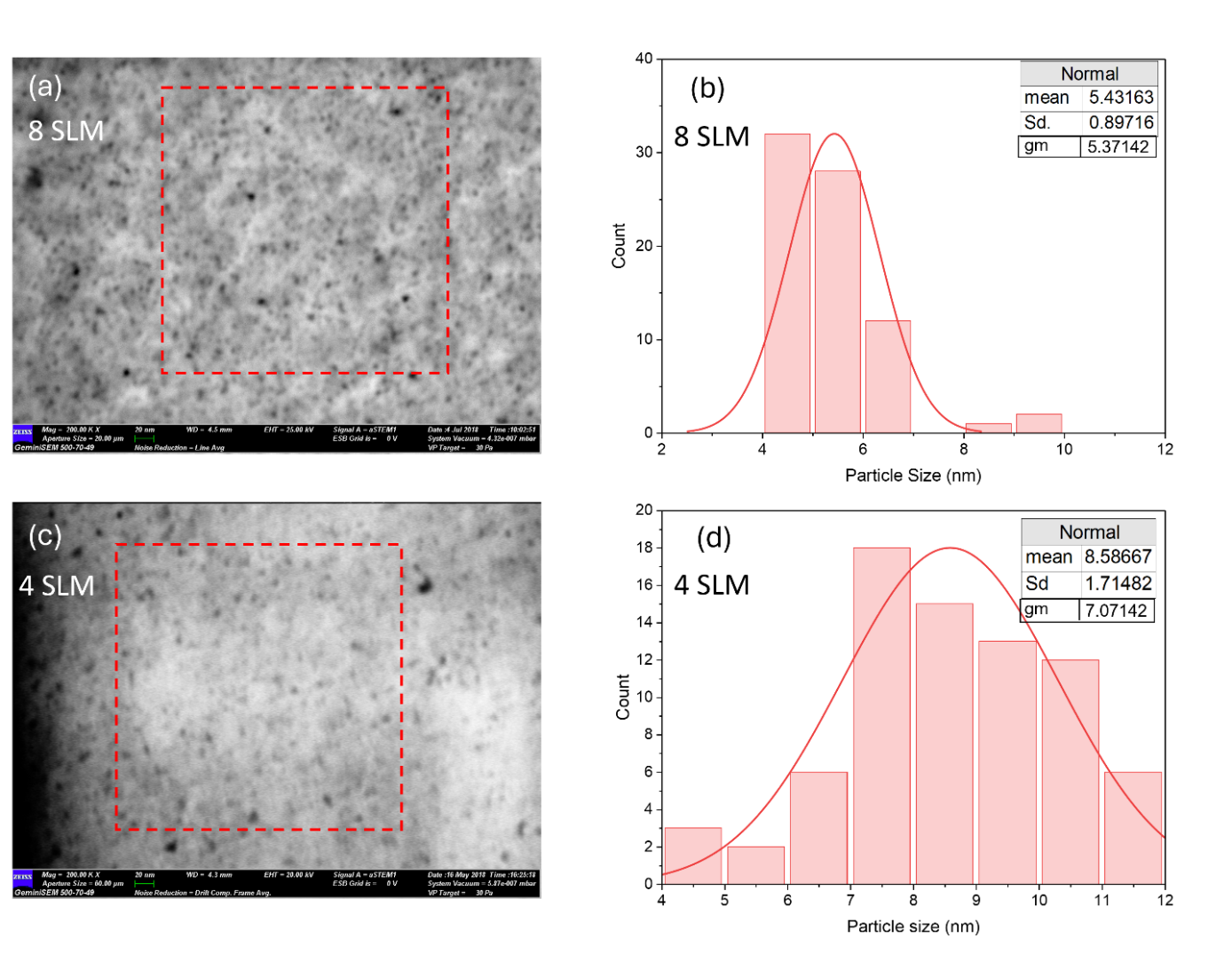


***Figure S1.1*** *STEM micrograph of Pt nanoparticles collected on a Cu grid substrate and 75 randomly selected particles are analyzed at (a) 8 standard liters per minute (SLM) and computation of mean, standard deviation and geometric mean are presented in (b). Similarly, another Cu grid is used to collect particles at (c) 4 SLM N_2_ flow rates and further analysed for mean, standard deviation and geometric mean in (d) for comparison.*

***Table S1*** *Particle size calculations for 75 randomly selected Pt nanoparticles for flowrates between 2 and 20 SLM*

**Supporting Information S2**

| **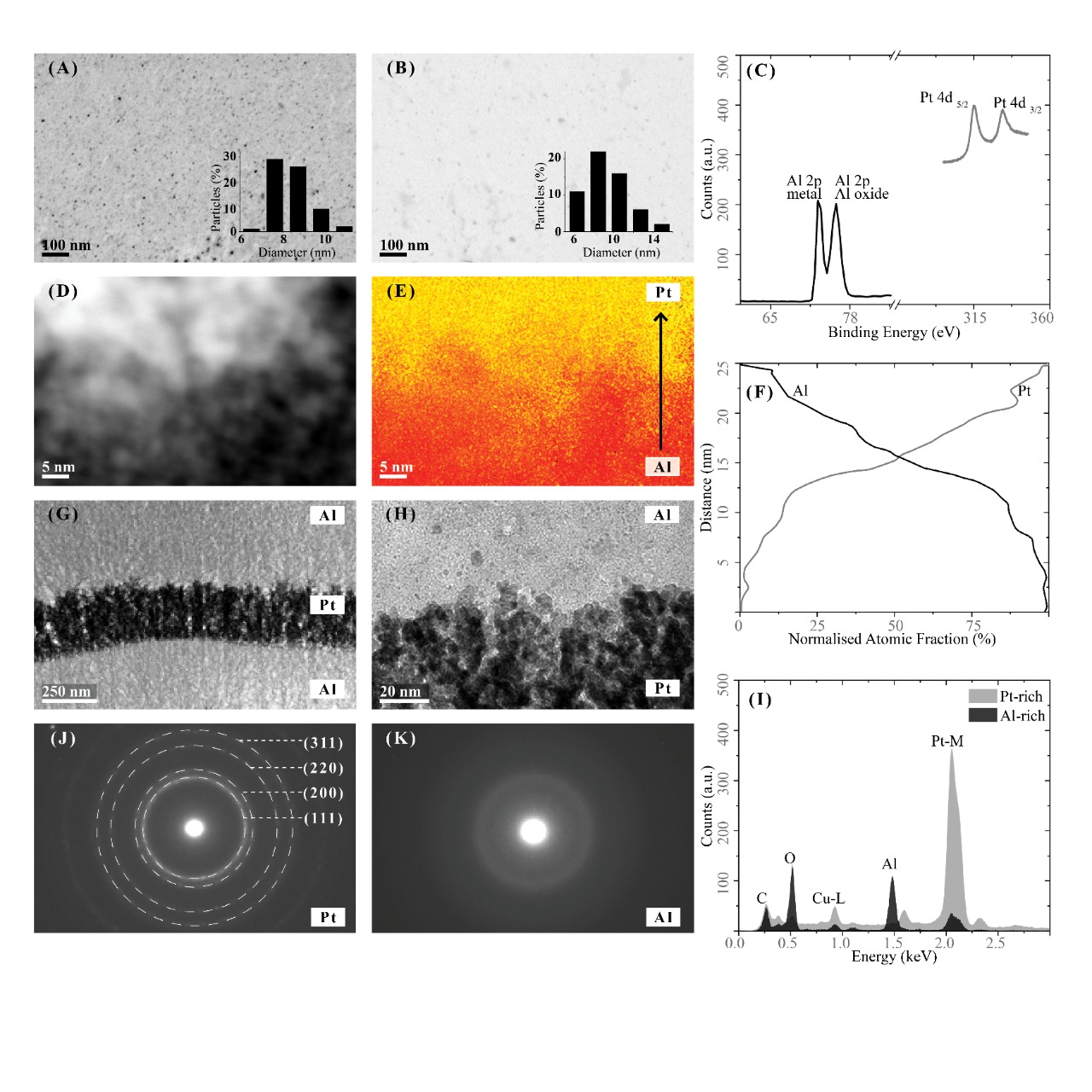** | **Figure S2.1** SAED rings obtained TEM measurements on a FIB lamella prepared and camera aperture focused on the Pt layer. The ring calculations are presented in Table S2. The concentric rings confirm that the Pt layer is polycrystalline. |
| --- | --- |

Under TEM imaging conditions, selected-area electron diffraction (SAED) patterns were registered at variable camera lengths (either 840 mm or 1.10 m), as exemplified in Figure S2.1. They show information from the material under observation in the reciprocal space, so the radii of the rings can be measured and eventually transformed into *d*-spacings in direct space. By looking up the values for several (*hkl*) planes in PDF file 04-0802, the rings can be indexed to various lattice planes belonging to Pt, which is polycrystalline in this case (Table S2).

**Table S2** The d-spacing calculations for Pt SAED concentric rings.

**Supporting Information S3**

Using Eje-Z software from University of Cádiz, we could analyse local areas by measuring FFT patterns retrieved from HRTEM micrographs. The resulting hexagonal arrangements of bright spots were fully measured (i.e. distances and angles) and subsequently compared with simulations in the aforementioned software. By comparing this experimental information to the calculations done for several PtAl intermetallics as cited in the manuscript, various potential similarities could be found. In Figure S3, the area of interest is shown as well as three exemplary FFT patterns with three possible simulation results given by Eje-Z software including angles and distances.


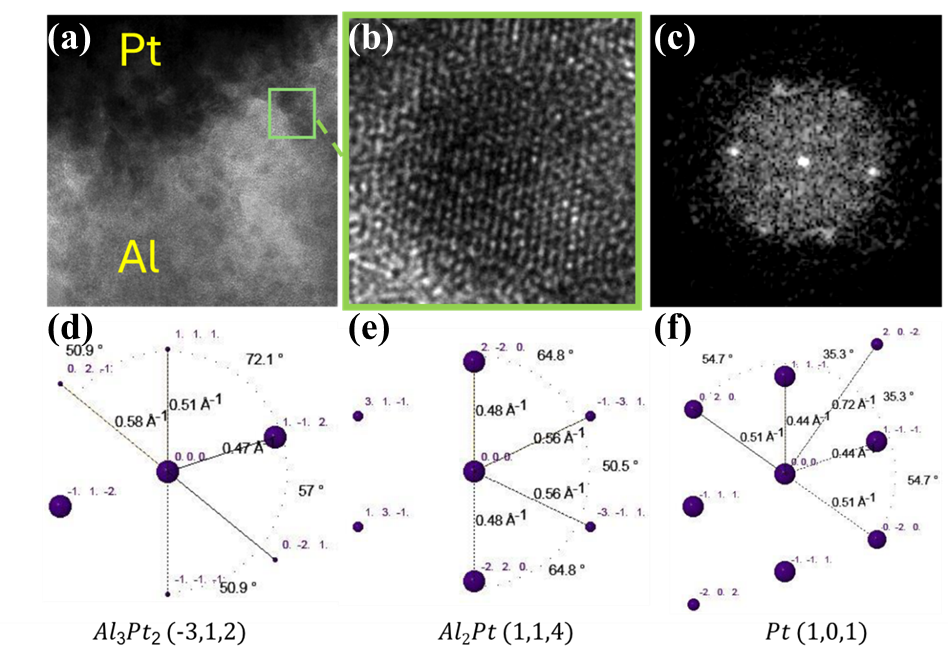


**Figure S3**. HRTEM results at the interfacial regions between Pt and Al to ascertain the formation of premixed layers. In (a) the area where HRTEM is carried out is shown. Another HRTEM image at higher magnification is shown in (b). Once FFTs are calculated and retrieved from these areas, hexagonal arrangements of bright spots can be seen (c), which are compared to results obtained by Eje-Z, as shown with three examples including (d) Al_3_Pt_2_, (e) Al_2_Pt and Pt (f).

**Supporting Information S4**

Post-reaction FIB cuts are performed on the reacted layer (Figure S3). As can be seen the multilayer stack cannot be seen anymore due to the formation of Al_2_Pt alloy. The morphology is rounded due to the intermediate liquid state which makes the surface morphology surface-tension dominated.

**
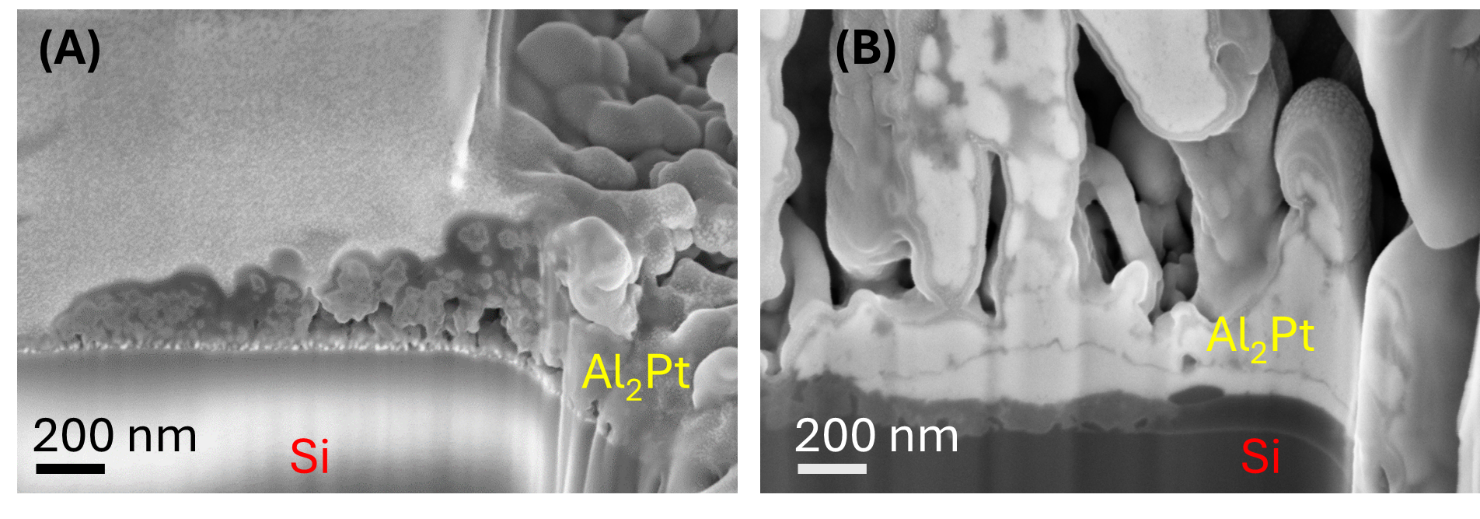
**

**Figure S4.** SEM micrographs of post-reaction FIB cuts. For FIB cuts, the linear deposition with reacted material is inserted into the machine and cross sections are observed. In (A), a 45º view is presented to have a look at the top surface of the deposition which shows rounded morphologies. In (B) the cross section shows a complete porous structure with curved morphology. The surface is rounded due to intermediate liquid phase.

**Supporting Information S5**

Reaction velocities were recorded for 30 different samples which belong to three distinct categories:

- Porous – spark power 2 W and carries gas flow rate 4 slm were used to make high porosity Pt films.
- Moderately dense – spark power 4 W and carries gas flow rate 6 slm were used to make intermediate porosity Pt films.
- Dense – spark power 4 W and carries gas flow rate 10 slm were used to make densely packed Pt films.

As the films become less porous, the probability of forming premixed regions at the interfaces between Pt-Al and the incorporation of air gaps decrease. This has a direct correlation to the reaction kinetics. With the increase in density, the kinetics are faster as can be seen in Figure S5.


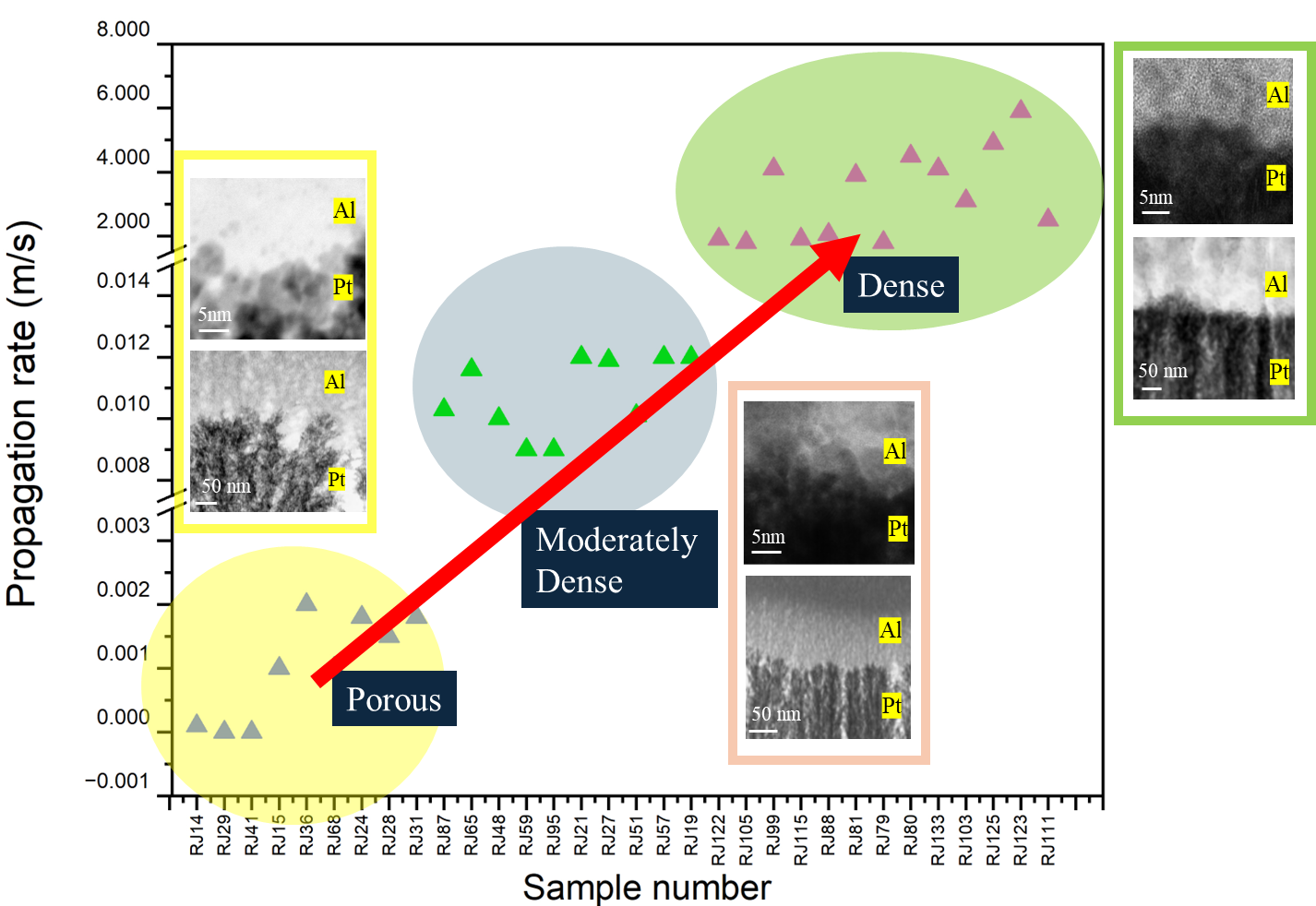


**Figure S5**. Three categories (Porous (yellow), moderately dense (blue) and dense (green)) of packing density of Pt films were deposited onto Si substrates. The number of bilayers and thickness was constant across reactions and the stacks were ignited. The reaction velocities are plotted for 30 different samples.
